# Prognostic significance of cytoplasmic S100A2 overexpression in oral cancer patients

**DOI:** 10.1186/s12967-014-0369-9

**Published:** 2015-01-16

**Authors:** Manish Kumar, Gunjan Srivastava, Jatinder Kaur, Jasmeet Assi, Akram Alyass, Iona Leong, Christina MacMillan, Ian Witterick, Nootan Kumar Shukla, Alok Thakar, Ritu Duggal, Ajoy Roychoudhury, Mehar Chand Sharma, Paul G Walfish, Shyam Singh Chauhan, Ranju Ralhan

**Affiliations:** Department of Biochemistry, All India Institute of Medical Sciences, New Delhi, 110029 India; Alex and Simona Shnaider Laboratory of Molecular Oncology, Mount Sinai Hospital, Toronto, Ontario Canada; Department of Clinical Epidemiology and Biostatistics, McMaster University, Hamilton, Canada; Department of Pathology & Laboratory Medicine, Mount Sinai Hospital, Joseph & Wolf Lebovic Health Complex, 600 University Avenue, Toronto, Ontario M5G 1X5 Canada; Department of Oral Pathology and Oral Medicine, Faculty of Dentistry, University of Toronto, 124 Edward Street, Toronto, Ontario M5G 1G6 Canada; Department of Otolaryngology – Head and Neck Surgery, Joseph and Mildred Sonshine Family Centre for Head and Neck Diseases, Mount Sinai Hospital, 600 University Avenue, Toronto, Ontario M5G 1X5 Canada; Department of Otolaryngology – Head and Neck Surgery, University of Toronto, 190 Elizabeth Street, Toronto, Ontario M5G 2N2 Canada; Department of Surgery, Dr. B. R. A. Institute Rotary Cancer Hospital, All India Institute of Medical Sciences, New Delhi, India; Department of Otorhinolaryngology, All India Institute of Medical Sciences, New Delhi, India; Centre for Dental Education and Research, All India Institute of Medical Sciences, New Delhi, India; Department of Medicine, Endocrine Division, Mount Sinai Hospital and University of Toronto, Toronto, Ontario M5G 1X5 Canada

**Keywords:** S100A2, Oral lesion, Squamous cell hyperplasia, Dysplasia, Squamous cell carcinoma, Head-and-neck cancer, Prognosis

## Abstract

**Background:**

Oral squamous cell carcinoma (OSCC) patients are at high risk of loco-regional recurrence and 5-year survival rates are about 50%. Identification of patients at high risk of recurrence will enable rigorous personalized post-treatment management. Most novel biomarkers have failed translation for clinical use because of their limited successful validation in external patient cohorts. The aim of this study was to determine the prognostic significance of alterations in sub-cellular expression of S100A2, a pro-tumorigenic calcium binding protein, identified as a candidate biomarker in our proteomic analysis in OSCC and validation of its clinical utility in an external cohort.

**Methods:**

In a retrospective study, immunohistochemical analysis of S100A2 was carried out in 235 Indian OSCC (Test set) and 129 normal oral tissues, correlated with clinicopathological parameters and disease outcome over 122 months for OSCC patients following the REMARK criteria. The findings were validated in an external cohort (Validation set 115 Canadian OSCC and 51 normal tissues) and data analyzed using the R package.

**Results:**

Significant increase in cytoplasmic and decrease in nuclear S100A2 expression was observed in OSCC in comparison with normal tissues. Cox multivariable regression analysis internally and externally validated cytoplasmic S100A2 association with tumor recurrence. Kaplan Meier analysis of patients stratified to high and low risk groups showed significantly different recurrence free survival (Test set- log rank test, p = 0.005, median survival 16 and 69 months respectively and Validation set - p < 0.00001, median survival 9.4 and 59.9 months respectively); 86% and 81% of patients who had recurrence were correctly stratified into the high risk group. Seventy percent and 81% patients stratified into low risk group did not show cancer recurrence within 1 year in Test and Validation sets.

**Conclusions:**

Our study provided clinical evidence for the potential of cytoplasmic S100A2 overexpression as a predictor of recurrence risk in OSCC patients. A unique translational aspect of our study is validation of S100A2 as prognostic marker in two independent cohorts (Canadian and Indian) suggesting this protein is likely to find widespread utility in clinical practice for identifying oral cancer patients at high risk of disease recurrence.

## Background

Oral squamous cell carcinoma (OSCC) is the tenth most prevalent cancer accounting for almost 300,000 new cases annually worldwide [1]. OSCC is the major subtype of head and neck squamous cell carcinoma (HNSCC) and accounts for two-thirds of the cases occurring in least developed countries [2]. OSCCs are often preceded by development of clinically distinct oral lesions; on an average about one percent of oral lesions transform into cancer annually [3,4]. Despite improvements in treatment strategies the prognosis of these patients remains largely unsatisfactory, due to loco-regional recurrence. The 5-year survival rates are about 50%, and the prognosis of advanced cases has not improved much over the past 4 decades [2]. At present, the most important prognostic factors include histological tumor grade, stage, depth of tumor invasion and involvement of regional lymph nodes at the time of diagnosis. In search of biomarkers of diagnostic or prognostic relevance, tissue proteomic analysis of OSCC using iTRAQ labeling liquid chromatography-mass spectrometry led to the identification of differential expression of S100A2 in OSCC in comparison with histologically normal oral mucosa [5,6].

S100A2, an 11.4 kDa protein, is a member of the S100 family of calcium-binding proteins that have diverse functions regulating a variety of cellular processes such as differentiation, regeneration, cell growth, and signal transduction in neoplastic cells [7]. S100A2 is distributed in the nucleus and cytoplasm of keratinocytes in normal human epidermis and is a component of the epidermal differentiation complex. The functional status of S100A2 protein was determined by interaction with other proteins, homodimers with S100 family members or heterodimers with other proteins in particular, with p53 and p63 [8]. Expression of S100A2 protein was found to be deregulated in a variety of tumor types. Initially diminished expression of S100A2 protein was reported in lung and gastric cancers [9-11]. However, several later studies have shown overexpression of S100A2 in other cancers including non-small cell lung cancer, esophageal, ovarian, bladder, breast, thyroid, melanoma and pancreatic cancer [12-19]. Recent studies reported S100A2 protein is a molecular driver in TGF-β induced cell invasion and migration in hepatic carcinoma [20]. Moreover, ectopic overexpression of S100A2 induces lung metastasis in mice [21]. Despite these reports, the clinical relevance of S100A2 expression as a prognostic marker for oral cancer patients remains to be determined.

We reported overexpression of S100A2 in OSCCs in proteomic analysis performed using isobaric mass tags for relative and absolute quantitation (iTRAQ) followed by multidimensional liquid chromatography/tandem mass spectrometry [5,6]. We hypothesized that alterations in S100A2 sub-cellular localization in cytoplasm or nucleus could influence oral cancer pathogenesis and may correlate with clinical outcome in these patients. In this study, we determined the clinical significance of alterations in expression and sub-cellular localization of S100A2 protein in OSCC by immunohistochemical analysis in an independent cohort of patients by comparing its expression to normal oral epithelium and determining its correlation with clinico-pathological parameters and disease outcome over 122 months to investigate its utility as a prognostic marker for OSCC. The clinical applicability of S100A2 as a prognostic marker in OSCC was validated in an external cohort. A unique translational aspect of our findings is the validation of S100A2 as a prognostic marker in two independent cohorts (Canadian and Indian) suggesting this protein is likely to find widespread utility in clinical practice for identifying oral cancer patients at high risk of disease recurrence.

## Materials and methods

### Study design

**Figure 1:**
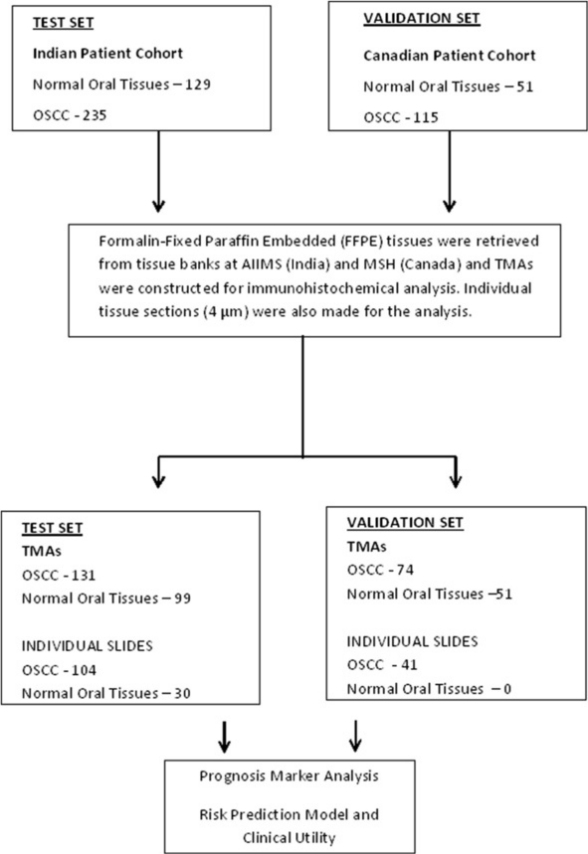
**Schematic diagram showing the study design.**

This study has been conducted in two institutions, Mount Sinai Hospital (MSH), Toronto, Canada and All India Institute of Medical Sciences (AIIMS), New Delhi, India according to the Reporting Recommendations for Tumor Marker prognostic Studies (REMARK) guidelines and a retrospectively written research, pathological evaluation, and statistical plan [22]. The study design is given in Figure 1. We have obtained appropriate consent and the retrospective studies were approved by the relevant research ethics boards prior to commencement.

### Patients

Patient demographic, clinical, and pathological data were recorded in a pre-designed Performa as described previously [23]. The information documented included clinical TNM staging (tumor, node, metastasis based on the Union International Center le Cancer TNM classification of malignant tumors), site of the lesion, histopathological grade, age, gender and treatment.

*Inclusion criteria*: Patients with histopathological evidence of squamous cell carcinoma of the oral cavity and a known clinical outcome were inducted into the study.

*Exclusion criteria*: Patients diagnosed with squamous cell carcinoma of the oral cavity but with no available follow-up data or patients diagnosed with dysplasia concomitant with OSCC at the first visit were excluded from the study.

### Test set. Specimen characteristics

Following the above inclusion and exclusion criteria, archived formalin fixed paraffin embedded (FFPE) tissue specimens were obtained from 235 OSCC patients (median age = 49 years; range - 19–85 years) undergoing curative cancer surgery during the period 2004 – 2011 in Department of Otorhinolaryngology, AIIMS. Wherever possible histologically normal tissues (n = 88) were taken from a site at least 5 cm distant from the surgically resected tumors from OSCC patients. Non-malignant normal oral tissues (n = 41) were also collected from the patients attending the Outpatient Department of Dental Surgery for tooth extraction. Taken together, these 129 oral tissues with histological evidence of normal epithelium constituted the normal group. After excision, tissues had been immediately snap frozen in liquid nitrogen and stored at −80°C in the Research Tissue Bank till further use; one part of the tissue had been collected in 10% formalin and embedded in paraffin for histopathological and immunohistochemical analysis. Histologically confirmed oral normal epithelia and OSCCs as revealed by H&E staining were used for immunohistochemistry [24]. These cases comprised the Test set.

### Validation set. Specimen characteristics

The patients’ charts with clinico-pathological diagnosis of OSCC from 2000 to 2010 were retrospectively reviewed to obtain the clinical information and follow-up data in the Department of Pathology, MSH. Information regarding gender, age, site of lesions at the time of the initial diagnosis of dysplasia or OSCC was documented in the clinical database. Following the above inclusion and exclusion criteria, archived tissue specimens of OSCC patients (n = 115, median age: 62 years; range: 21–92 years) undergoing curative cancer surgery during the period 2000 – 2010 were inducted into this study and 51 paired normal tissues were also obtained from the archived tissue bank at MSH, Canada.

### Treatment

All OSCC patients were treated as per the National Comprehensive Cancer Network (NCCN) guide lines for head and neck cancers (www.nccn.org). OSCC patients with T_1_ and T_2_ tumors were treated with radical surgery; majority of patients with T_3_ and T_4_ disease were treated with radical surgery followed by postoperative radical radiotherapy [23].

### Survival data

After completion of the primary treatment OSCC patients were followed at regular time intervals in both Centers AIIMS and MSH for a maximum period of 122 months (mean 23.08 months and median 11.50 months). Notably, recurrence or death was observed in 51.1% patients. The medical history, clinical examination, and radiological evaluation were used to determine whether the death had resulted from recurrent cancer (relapsing patients) or from any other causes. Disease-free survivors were defined as patients free from clinical and radiological evidence of local, regional, or distant relapse at the time of the last follow-up. In the current study, recurrence of the cancer and/or death versus no recurrence of OSCC was considered to be the clinical outcome of the patients. Follow-up period was defined as the interval from the time when patient underwent first surgery to recurrence of cancer or death (for uncensored observations) or no recurrence at last consultation (for censored observations).

### Assay methods

#### Histopathology, tissue microarrays (TMAs) construction and immunohistochemistry

The histopathologic diagnosis of all cases were re-examined and confirmed by the oral pathologists at MSH and AIIMS respectively. The tissue sections comprising of more than 70% epithelial cells (cancer/normal) were selected for further analysis using immunohistochemistry. Tissue microarrays (TMAs) were constructed using 131 of 235 OSCCs and 99 of 129 normal oral tissues analyzed in Test set and 74 of 115 OSCCs and 51 normal oral tissues in Validation set, while the remaining tissues were used as individual sections for immunostaining as described below. The construction of TMAs was recently reported [25]. The TMA blocks were constructed by relocating small cylindrical tissue cores (two cores per tissue block representing the cancer sections) from individual donor blocks and placing them in a recipient block with defined array coordinates. Arrays were constructed from FFPE tissues by the removal of 0.6 mm diameter tissue cores from donor blocks. A total of two morphologically representative areas of interest from each donor block were identified under the microscope by the pathologists using a stained H&E section as a guide. Using a precise spacing pattern on manual TMA instrument, 150–200 cores could be transferred to the recipient paraffin block in a grid like fashion, retaining a link to the original block and its pathology. Consecutive 4 μm sections were cut from the recipient block and used for immunohistochemical staining for S100A2 protein.

Paraffin-embedded TMA sections or individual tissue sections (4 μm) of human oral non-malignant tissues and OSCCs collected on gelatin-coated slides were used for immunostaining as described [25]. In brief, the sections were deparaffinized in xylene, hydrated in gradient alcohol followed by antigen retrieval. The sections were incubated with hydrogen peroxide (0.3% v/v) to quench the endogenous peroxidase activity, followed by blocking with 1% bovine serum albumin (BSA) to preclude non-specific binding. Thereafter, the slides were incubated with rabbit monoclonal anti-S100A2 antibody (0.5 μg/ml, [EPR5392] (ab109494, Abcam, CA) for 16 h at 4°C. The primary antibody was detected using the Dako Envision kit (Dako CYTOMATION, Glostrup, Denmark) and diaminobenzidine as the chromogen [6]. Slides were washed with Tris-buffered saline (TBS, 0.1 M, pH = 7.4), 3–5 times after every step. Finally, the sections were counterstained with Mayer’s hematoxylin and mounted with D.P.X mountant. In the negative control tissue sections, isotype specific non-immune rabbit IgG replaced the primary antibody. Cervical cancer tissue sections were used as a positive control for S100A2 expression. The sections were evaluated by light microscopic examination using Olympus BX51 microscope (Olympus, NY). Two researchers from AIIMS (MK, JK) were trained in MSH for Tissue microarrays (TMAs) construction, immunohistochemical staining and scoring to ensure use of identical methodologies.

### Evaluation of immunohistochemical staining

Each TMA slide or individual tissue section was evaluated for S100A2 immunoreactivity using a semi-quantitative scoring system for both staining intensity and the percentage of positive epithelial cells as described [25]. Immunopositive staining was evaluated in randomly selected five areas of the tissue section. For S100A2 protein expression, sections were scored as positive if epithelial cells showed immunostaining in the nucleus/cytoplasm when observed independently by three of us, who were blinded to the clinical outcome (the slides were coded and the scorers did not have prior knowledge of the local tumor burden, lymphonodular spread, and grading of the tissue samples). The tissue sections were scored based on the % of immunostained cells as: 0–10% = 0; >10–30% = 1; >31–50% = 2; >51–70% = 3 and >71–100% = 4. Sections were also scored semi-quantitatively on the basis of staining intensity as negative = 0; mild = 1; moderate = 2; intense = 3. Finally, a total score was obtained by adding the score of percentage positivity and intensity therefore giving a score range from 0 to 7 [25].

### Statistical analyses

Statistical analyses were carried out using R version 3.01 (http://www.r-project.org/). The relationships between S100A2 expression and patients’ characteristics were compared using Kruskal-Wallis rank sum tests. Nested Cox regression LRTs were used to guide variable selection between subcellular % positivity and intensity of S100A2 immunostaining. LRTs were used to assess whether: 1) cytoplasm and nucleus variables added independent information upon each other that improve the model fit; and 2) intensity added significant improvement to model fit upon their respective nuclear or cytoplasmic % positivity. Thus the most significant subcellular variables of S100A2 were inferred. Univariate and multivariable Cox regression analyses were used to assess the prognostic value of S100A2 alone and independent of clinical and pathological parameters (tumor stage, nodal status, and histology grade). Two-sided p values were calculated and p < 0.05 was considered to be significant. Regression estimates in the Test set (Indian) and Validation set (Canadian) were compared to assess the stability of S100A2 as a prognostic factor for cancer recurrence/death. All model performances were compared using Harrell’s c-statistic [26]. Internal validations based on 9999 bootstrap samples were used to address potential overfitting issues, and to correct for optimism. Cox proportional hazards assumption was assessed graphically and tested using the chi-squared test for goodness of fit on Schoenfeld residuals [27]. Cox proportional hazard models were fitted using *rms* package in R [28]. S100A2 median value derived from the Test sample set was used to classify subjects into high and low risk groups for recurrence. Kaplan-Meier plots were used to compare the survival curves of the high and low risk groups. Time-dependent Area Under the Curve (AUC) plots were used to assess improvements of S100A2 upon common clinical parameters. This was done by comparing the time-dependent AUC of a baseline model using clinical parameters alone, and an extended baseline model with both clinical parameters and S100A2. The clinical relevance of S100A2 to stratify patients into high and low risk groups for cancer recurrence/death was assessed using sensitivity, specificity, Positive Predictive Value (PPV), and Negative Predictive Value (NPV), and the AUC of the Receiver Operating Characteristics (ROC) curve. The same cut-off value was further validated in the Validation set.

## Results

### Immunohistochemical analysis of S100A2 expression in OSCC

**Figure 2:**
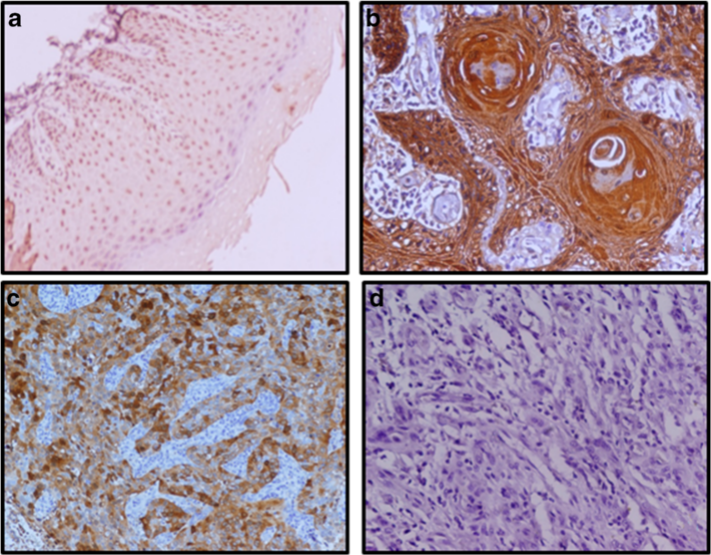
**Immunohistochemical analysis of S100A2 in oral tissues.** Paraffin-embedded sections of histologically normal mucosa and OSCC were stained using a rabbit monoclonal anti-S100A2 antibody (0.5 μg/ml, [EPR5392] (ab109494, Abcam, CA) as described in Material & Methods section. Panel represents **(a)** normal oral mucosa showing nuclear S100A2 immunostaining; **(b)** OSCC section illustrating cytoplasmic S100A2 and loss of nuclear staining in tumor cells; **(c)** Cervical cancer tissue section showing cytoplasmic S100A2 immunostaining; **(d)** OSCC section used as a negative control, showing no S100A2 immunostaining in tumor cells; (original magnification x 200).

To determine the clinical significance of S100A2 protein in oral cancer, its expression was analyzed in OSCC and histologically normal tissues using a specific monoclonal antibody by immunohistochemistry. Nuclear and cytoplasmic staining was scored based on the percentage of immunopositive cells and intensity of staining. The results of % positivity of the respective sub-cellular localization (nuclear and cytoplasmic) are summarized in Table 1. Figure 2a, b show representative photomicrographs of S100A2 immunostaining in normal oral tissue and OSCC respectively. Cervical cancer used as a positive control showed nuclear and cytoplasmic expression of S100A2 protein (Figure 2c), while no immunostaining was observed in tissue sections used as negative controls where the primary antibody was replaced by isotype specific IgG (Figure 2d). Significant increase in cytoplasmic S100A2 expression was observed in OSCC as compared to the normal oral tissues p < 0.0001, Test set and p = 0.05, Validation set, Table 1). In comparison, significant loss of nuclear S100A2 expression was observed in OSCC as compared to the normal oral tissues p = 0.02, Test set and p = 0.04, Validation set, Table 1). Importantly, cytoplasmic S100A2 showed significant correlation with disease recurrence (p < 0.01, Test set and p = 0.05, Validation set, Table 1).

**Table 1:** **Analysis of S100A2 protein expression and correlation with clinicopathological parameters**

**Clinicopathological**	**Test set**				**External validation set**			
**Features**	**N**	**Cytoplasmic**	**Nuclear**	**p**	**N**	**Cytoplasmic**	**Nuclear**	**p**
		**Mean (SD)**	**Mean (SD)**			**Mean (SD)**	**Mean (SD)**	
**Normal**	129	1.29 (0.99)	1.07 (1.29)		51	2.55 (0.88)	2.64 (1.05)	
**OSCC**	235	3.04 (1.26)	0.75 (1.03)	<0.0001^1^	115	2.72 (1.06)	2.18 (1.13)	0.05^1^
				0.02^2^				0.04^2^
**Age (Median, 49 years)**								
<49	115	2.97 (1.31)	0.75 (1.02)	0.40^1^	17	3.12 (1.17)	2.50 (1.17)	0.05^1^
≥49	120	3.12 (1.20)	0.75 (1.05)	0.86^2^	98	2.70 (1.01)	2.17 (1.12)	0.22^2^
**Gender**								
Male	184	3.05 (1.26)	0.73 (1.01)	0.65^1^	68	2.74 (1.03)	2.24 (1.10)	0.68^1^
Female	51	3.00 (1.26)	0.80 (1.11)	0.90^2^	47	2.81 (1.06)	2.18 (1.17)	0.73^2^
**Differentiation**								
WDSCC	115	3.08 (1.27)	0.81 (1.08)		28	2.82 (1.06)	2.54 (1.10)	
MDSCC	113	2.97 (1.27)	0.70 (0.99)	0.46^1^	70	2.86 (0.92)	2.22 (1.08)	0.28^1^
PDSCC	7	3.57 (0.53)	0.57 (0.98)	0.73^2^	17	2.29 (1.36)	1.68 (1.24)	0.06^2^
**Clinical stage**								
I	3	2.67 (2.31)	0.67 (1.15)		30	2.70 (1.29)	2.32 (1.26)	
II	38	2.79 (1.26)	0.79 (1.17)		21	2.81 (0.75)	2.48 (1.12)	
III	69	3.12 (1.18)	0.65 (0.87)	0.36^1^	21	2.52 (1.03)	1.67 (0.91)	0.60^1^
IV	125	3.09 (1.28)	0.79 (1.08)	0.94^2^	43	2.91 (0.97)	2.29 (1.07)	0.09^2^
**Tumor stage**								
I	6	2.67 (1.51)	1.17 (1.33)		48	2.77 (1.17)	2.25 (1.18)	
II	69	2.84 (1.26)	0.81 (1.07)		38	2.53 (0.95)	2.04 (1.22)	
III	65	3.20 (1.19)	0.65 (0.89)	0.14^1^	18	3.06 (0.80)	2.28 (0.89)	0.08^1^
IV	95	3.11 (1.28)	0.75 (1.08)	0.71^2^	10	3.30 (0.67)	2.75 (0.72)	0.37^2^
**Nodal status**								
N_0_	81	2.86 (1.43)	0.68 (0.99)	0.32^1^	63	2.78 (1.04)	2.35 (1.11)	0.88^1^
N_1–4_	154	3.14 (1.15)	0.79 (1.06)	0.46^2^	52	2.75 (1.05)	2.06 (1.13)	0.21^2^
**Recurrence status** ^**3**^								
Non-recurrent	82	2.60 (1.39)	0.80 (1.04)	<0.01^1^	47	2.60 (0.92)	2.23 (1.14)	0.05^1^
Recurrent	80	3.30 (1.07)	0.89 (1.13)	0.80^2^	55	2.91 (1.11)	2.16 (1.10)	0.79^2^

^1^p of Kruskal-Wallis rank sum test on cytoplasmic S100A2 % positivity score; ^2^p of Kruskal-Wallis rank sum test on nuclear S100A2 % positivity score; ^3^Only for OSCC cases with follow up information.

### Prognostic analyses of S100A2 in oral cancer

Nested Likelihood Ratio Tests (LRTs) of Cox regression models using the Test set indicated that cytoplasmic % positivity of S100A2 was the most predictive attribute for time of recurrence/death (Table 2). Cytoplasmic S100A2 % positivity was found to be associated with poor disease prognosis in univariate analysis [Hazard Ratio HR = 1.35 (95% CI = 1.09, 1.67), p < 0.01, Table 3]. These results were internally validated based on 9999 bootstrap samples (Table 3). External validation was carried out using an independent cohort of Canadian patients (validation set). The validation data set showed similar effect sizes and association levels of S100A2 with disease prognosis [HR = 1.40 (95% CI = 1.04, 1.90), p = 0.03, Table 3] confirming the applicability of cytoplasmic S100A2 % positivity as a prognostic factor for cancer recurrence/death. Multivariable Cox regression analyses confirmed the prognostic value of S100A2 independent of clinicopathological parameters [HR = 1.33 (95% CI = 1.07, 1.65), p = 0.01, Table 3]. Internal and external validations of these findings gave similar results (Table 3). Loss of nuclear S100A2 expression did not correlate significantly with disease prognosis.

**Table 2:** **Variable selection using nested LRT in Test set**

**Fitted Cox models**	p*
Nuclear vs. Cytoplasmic S100A2 attributes	
Nuclear variables	0.70
Cytoplasmic variables	<0.01
Combined nuclear and cytoplasmic variables	0.04
Nested LRT	0.34
Most predictive subcellular variables	Cytoplasmic S100A2
% Positivity alone vs. % Positivity & Intensity	
Cytoplasmic variables	<0.01
Without cytoplasmic intensity	<0.01
Nested LRT	0.07
Most predictive variable	cytoplasmic % positivity

*p corresponds to the LRT of the model fit, unless stated otherwise.

**Table 3:** **Cox regression analyses**

**Predictors**	**Test set (n = 162)**			Internal validation of test set*			**External validation set (n = 102)**		
	**HR [95% CI]**	**p**	**c-statistic**	**HR [95% CI]**	**p**	**c-statistic**	**HR [95% CI]**	**p**	**c-statistic**
Univariate analyses									
Cyto S100A2 % Pos	1.35 [1.09, 1.67]	<0.01	0.59	1.35 [1.10, 1.76]	0.01	0.59^€^	1.40 [1.04, 1.90]	0.03	0.60
Multivariable analyses adjusted for clinical parameters									
Cyto S100A2 % Pos	1.33 [1.07, 1.65]	0.01	0.62	1.34 [1.08, 1.78]	0.03	0.59^€^	1.47 [1.07, 2.03]	0.02	0.67
Histology grade	1.32 [0.91, 1.91]	0.14		1.32 [0.90, 1.92]	0.14		1.03 [0.65, 1.64]	0.90	
Clinical stage	1.06 [0.60, 1.89]	0.83		1.07 [0.59, 1.93]	0.84		1.00 [0.65, 1.54]	0.99	
Nodal status	1.30 [0.68, 2.51]	0.43		1.33 [0.69, 2.82]	0.45		2.09 [0.89, 4.92]	0.09	
Tumor stage	1.10 [0.72, 1.70]	0.65		1.11 [0.71, 1.77]	0.67		1.23 [0.86, 1.78]	0.25	

^*^Internal validations were based on 9999 bootstrap samples; ^€^Optimism corrected c-statistic.

### Time dependent prognostic analyses of S100A2 and clinical parameters

**Figure 3:**
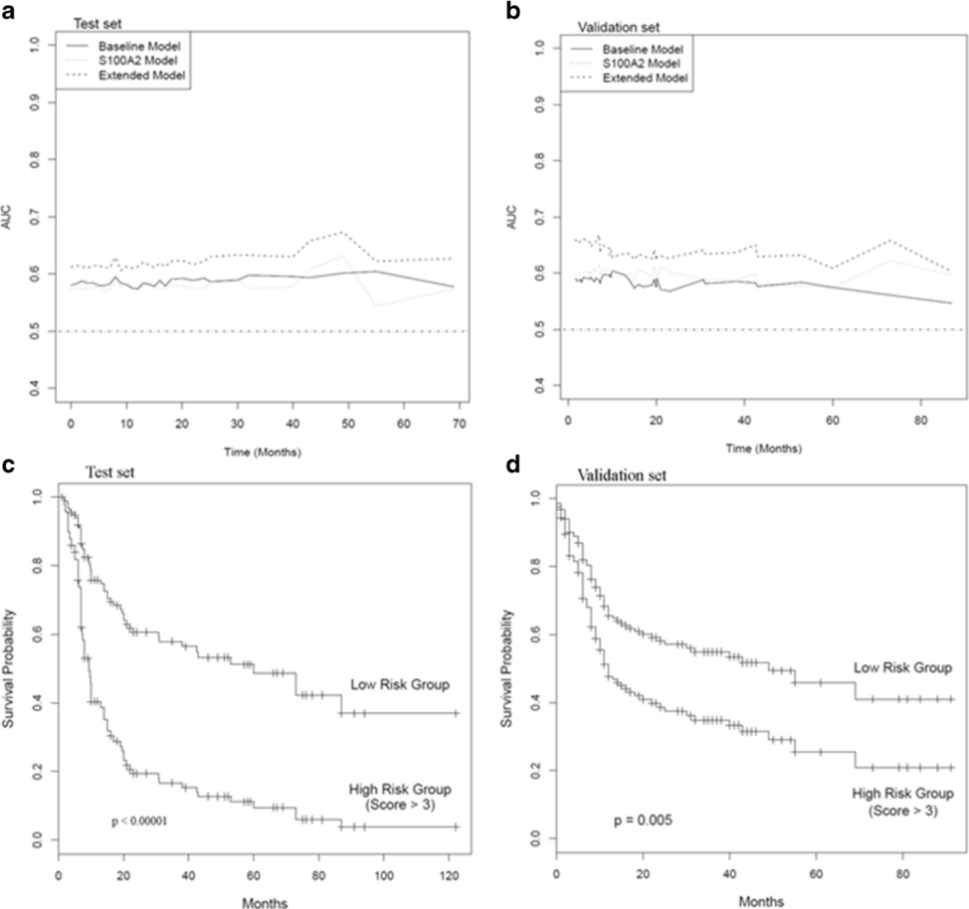
**Time-dependent AUC plots of cytoplasmic S100A2 in OSCC and evaluation of S100A2 overexpression as a prognostic marker of OSCC.** Time-dependent AUC plots for a baseline model (clinical parameters alone), a univariate model (cytoplasmic S100A2 % positivity) and an extended baseline model (clinical parameters + cytoplasmic S100A2 % positivity) in **a**. Test set and **b**. Validation set. **c**. Survival curves of the high risk and low risk groups in the Test set. Median survival times (months): High risk group - 16 (95% CI = 8.00, 40); Low risk group - 69 (95% CI = 14, −--). **b**. Survival curves of the high risk and low risk groups in the validation set. Median survival times (months): High risk group - 9.4 (95% CI = 7.00, 38.1); Low risk group - 59.9 (95% CI = 21, −--).

The time-dependent AUC plots for the baseline models (clinical parameters alone), univariate models (cytoplasmic S100A2 % positivity) and the extended baseline models (clinical parameters + cytoplasmic S100A2 % positivity) in both sample sets confirmed that S100A2 together with clinical parameters hold better discriminatory ability throughout time compared to the use of clinical parameters alone (Figure 3a, Figure 3b). These results confirm the independent contribution of S100A2 and in combination with clinical parameters in assessing disease prognosis.

### Clinical Utility of S100A2

The median cytoplasmic S100A2 % positivity in OSCCs in Test set was used to classify patients into high (% positivity score >3) and low (% positivity score ≤ 3) risk groups. Kaplan Meier analysis of patients stratified to high and low risk groups showed significantly different survival probabilities (log rank test, p < 0.00001, Figure 3c). The median survival time of the higher risk group was found to be 16 months (95% CI = 8.00, 40). This is compared to a median time survival of 69 months with a 95% CI lower bound of 14 months. The same cut-off value was used to stratify patients in the validation set into high and low risk groups. Kaplan Meier analysis of patients stratified to the high and low risk groups in the validation set also showed significantly different survival probabilities (log rank test, p = 0.005, Figure 3d). The median survival time of the high and low risk groups were found to be 9.4 months (95% CI = 7.00, 38.1), and 59.9 months (95% CI = 21, −--) respectively. The consistency of findings in the test set and validation set confirmed that disease prognosis was significantly different between the high and low risk groups. The clinical utility of cytoplasmic S100A2 % positive score in predicting recurrence/death within 1 year was assessed in both sample sets. Using a cut-off value of 3, 86% and 81% of patients who had recurrence / death within 1 year were correctly stratified into the high risk group in the Test and Validation sample sets respectively. Furthermore, 70%, and 81% of subjects stratified into the low risk groups in both samples did not show cancer recurrence within 1 year in the Test and Validation sample sets respectively. The strength of S100A2 as a tool in clinical settings is mainly due to its ability to capture the recurrence cases correctly in both test and validation sets. Consequently, higher percentages of true negatives were stratified to the low risk group enhancing the negative predictive value of this stratification approach.

## Discussion

A major challenge is to predict the prognosis of OSCC patients effectively after completion of their primary treatment. In this context our study assumes importance, because of its retrospective nature, the large set of patients representing different stages of OSCC from two independent cohorts and long-term follow-up analysis. Our study uniquely based on sub-cellular compartment analysis of S100A2 expression for correlation with clinical outcome, gave a more comprehensive insight into the clinical relevance of alterations in sub-cellular localization of a protein on disease outcome. Interestingly, a subset of patients having OSCC showed increased cytoplasmic S100A2 expression and loss of nuclear S100A2 expression in these tissues as compared to normal tissues. Furthermore, the cytoplasmic overexpression of this protein in OSCCs was associated with disease recurrence. Hence, our study emphasizes the importance of sub-cellular compartmental analysis of S100A2 protein in cytoplasm and nucleus as compared to the overall protein expression reported in most earlier studies [19,29].

Our findings are supported by a recent study that demonstrated S100A2 is required for TGF-β induced cell migration and cell invasion [20]. These authors proposed that interaction of S100A2 with smads in the cytoplasm leading to protein stabilization is a necessary step for TGF-β induced tumorigenesis [20]. Bulk et al., (2009) reported S100A2 overexpression induced migration, invasion and metastasis in lung cancer suggesting a central role for this protein in proliferation pathways, and its potential to serve as a therapeutic target for treatment of inflammation and cancer. Tissue microscopic examination has shown higher expression of S100A2 predominantly in the proliferative layer suggesting S100A2 may be a molecular player during the inflammatory cell response and disease progression, a role supported by the finding that overexpression of S100A2 attracts inflammatory eosinophils during immune response [21]. Importantly, recent demonstration of the pro-tumorigenic actions of S100A2 in lung cancer cells involving regulation of PI3K/Akt signaling and functional interaction with Smad3, which is enhanced in the presence of calcium and TGF-β and induces epithelial-mesenchymal transition (EMT) [20], further support our clinical findings of increased cytoplasmic and decreased nuclear S100A2 expression in OSCC. Loss of nuclear S100A2 has been reported in multiple cancers including early stage oral cancer, pancreatic cancer, non-small cell lung carcinoma [18] and our findings of nuclear loss of S100A2 support these studies. However, most studies did not investigate the clinical significance of cytoplasmic S100A2. Changes in sub-cellular localization of proteins often affect their normal cellular function. Using oral cancer cell lines, Mueller et al., showed that S100A2 protein translocated from the cytoplasm to the nucleus and co-localized with tumor suppressor p53 and increased its transcriptional activity thereby modulating cellular proliferation [8]. Our study underscores the biological relevance of nuclear loss of S100A2 expression and its cytoplasmic accumulation in oral cancer. Notably, cytoplasmic S100A2 expression emerged as a poor prognosticator in OSCC underscoring the clinical significance of S100A2 in oral cancer. The time-dependent AUC plots showed that S100A2 alone had comparable discriminatory ability to the clinical parameters throughout survival time. The unique aspect of our study is the validation of prognostic utility of S100A2 in an independent external cohort (Canadian) which showed similar association with disease outcome as observed in the test set comprising of Indian patients. The robustness of our findings was supported by internal validation of prognostic utility of S100A2 in the Test set by bootstrapping. Based on cytoplasmic S100A2 expression levels, the stratification of OSCC patients into high and low risk groups accurately predicted their disease outcome; 86% and 81% of patients who had recurrence/death within 1 year were correctly stratified into the high risk group in the Test and Validation sets respectively. Furthermore, 70% and 81% of subjects stratified into the low risk groups in both sets did not show cancer recurrence within 1 year respectively. The strength of S100A2 as a tool in clinical settings is mainly due to its ability to capture the recurrence cases accurately. Consequently, higher percentages of true negatives were stratified to the low risk group enhancing the negative predictive value of this stratification approach. One limitation of our study is that it does not provide mechanistic insight into the role of S100A2 in oral cancer. In conclusion, we demonstrate S100A2 is overexpressed in oral cancer. Importantly, cytoplasmic S100A2 emerged as an independent predictor of recurrence in OSCC patients suggesting its potential to serve as a prognostic marker in oral cancer patients.
